# Evaluation of volumetric measurements on CBCT images using stafne bone cavities as an example

**DOI:** 10.4317/medoral.20633

**Published:** 2015-06-27

**Authors:** Mehmet-Zahit Adisen, Selmi Y?lmaz, Melda Misirlioglu, Fethi Atil

**Affiliations:** 1 Lecturer (PhD). Department of Oral and Maxillofacial Radiology. Faculty of Dentistry, K?r?kkale University, K?r?kkale, Turkey; 2 Research Assistant (PhD). Department of Oral and Maxillofacial Radiology. Faculty of Dentistry, K?r?kkale University, K?r?kkale, Turkey; 3 Assistant Professor (PhD). Department of Oral and Maxillofacial Radiology. Faculty of Dentistry, K?r?kkale University, K?r?kkale, Turkey; 4 Assistant Professor (PhD). Department of Oral and Maxillofacial Surgery. Faculty of Dentistry, K?r?kkale University, K?r?kkale, Turkey

## Abstract

**Background:**

The aim of the present study is to evaluate the efficacy of CBCT in volume measuring using Stafne Bone Cavities (SBC) as an example.

**Material and Methods:**

The study was conducted with 14 subjects with SBC detected on panoramic radiographs. In order to evaluate lesions volumetric dimensions, CBCT images for each patient were captured. Files in Digital Imaging and Communications in Medicine (DICOM) format were transferred into a medical image processing program (ITK-SNAP 2.4.0) and volume in mm3 of the cavities were measured using semi-automatic segmentation procedure by 2 observers blinded to each other over a one-month period. Inter-reliability of volumetric measurements between observers was compared. SBCs relation to mandibular canal was also examined and three types of relation were observed; type 1: mandibular canal is separated from the SBC, type 2: mandibular canal is in contact with SBC, type 3: mandibular canal goes through the SBC.

**Results:**

There were 12 males and 2 females who had SBC in this study (age range: 37-73, mean age: 55.3 years). The total volume of SBC in patients ranged from 160 mm3 to 520 mm3 (mean: 361.7 mm3). There was no significant difference between observers for volume measurements (*p*>0.05). According to relationship of SBC with mandibular canal, most SBCs were Type 1 (64.3 %) followed by type 3 (21.4 %) and type 2 (14.3 %). Pearson correlation coefficient shows a positive correlation between lesions volumetric size and relation with mandibular canal (pearson correlation = 0.54, sig < 0.05).

**Conclusions:**

Based on the results of this preliminary study, CBCT was considered to be an effective radiographic technic for measuring volumetric sizes of SBCs. However further studies with larger sample sizes are needed to prove the usefulness of CBCT in volume measurements.

**Key words:**Stafne bone cavity, CBCT, volumetric measurements, image segmentation.

## Introduction

Volumetric measurements in the field of dentomaxillofacial radiology are usually made for detecting upper airway or sinonasal complex volume (1). They can also be used for evaluating volumetric size of various bone lesions including; periapical abscess, cysts and tumors (2). Detecting the volume of a lesion becomes important especially comparing the lesions dimensions with the follow-up radiographies. It is also important when reconstructive surgery with bone grafting is planned (3).

When surgical access is necessary, quality imaging is critical to localize the lesion and its proximity to important structures. While the information gleaned from a dental radiograph is substantial, there are limitations associated with the use of a two-dimensional image. Therefore, an imaging modality with three- dimensional (3D) capability is essential to enhance diagnosis and treatment (4). The introduction of cone beam computed tomography (CBCT) has initiated a new era in the field of dentomaxillofacial radiology owing to the acquisition of large data volume in a short scan-time and at low radiation dose. CBCT scans produce reconstructed multiplanar images that allow the clinician to assess the area of interest three-dimensionally. It provides clear images of high-contrasted structures and is extremely useful for evaluating bone pathologies (5).

Many studies investigating dimensional accuracy of CBCT quote linear or angular measurements and they usually employed methods using calibration cubes, spherical phantoms of known size, calculating volume from manual linear readings or simulated lesions (2,6,7). Currently, there are only a few studies confirming the accuracy of CBCT to measure lesions in bone volumetrically (5).

Stafne Bone Cavity (SBC) is a homogeneous radiolucent lesion with well-defined borders usually located in the mandible below the inferior dental canal, a suitable candidate for volumetric measurements on CBCT images. It was stated that CBCT provides accurate information on the shape, location and size of SBC (8). In the present study, we aimed to evaluate the efficacy of CBCT for volumetric measurement using SBCs as an example.

## Material and Methods

The study was conducted with 14 subjects selected from among 16782 patients who underwent clinical and panoramic radiographic examinations in the Department of Oral and Maxillofacial Radiology between January 2012 and October 2013. Panoramic radiographs were obtained using OP 200D (Instrumentarium Dental, Tuusula, Finland) by two radiography technicians both of whom had a minimum working experience of 5 years. (57-85 kVp, 2-16 mA, and 8 sec.)

Panoramic radiographs were examined by three oral radiologists and one oral surgeon who were able to use brightness and contrast tools to optimize their diagnosis and classify SBC on 14 cases using the same criteria under optimum viewing conditions. All of the patients agreed to participate in the study and signed an informed consent form. This study had ethical approval by the Research Committee of the Faculty of Dentistry at K?r?kkale University and is in compliance with the Helsinki Declaration.

An ID number has been assigned for every patient; who has been associated with collected data pertaining to demographic information (age, sex), medical and dental history, and the location of the cavity.

In order to evaluate SBCs volumetric dimensions, CBCT images for each patient were captured using PaxUni 3D (Vatech, Seoul, Korea) at the following settings: 50-90 kVp, 4-10 mA, 10 sec exposure time, and a 50×50 mm field of view (FOV) size. Obtained image files in Digital Imaging and Communications in Medicine (DICOM) format were transferred into a medical image processing program (ITK-SNAP 2.4.0) and volume in mm3 of the cavities were measured using semiautomatic segmentation procedure (0.2-mm slice thickness).

**Figure 1:**
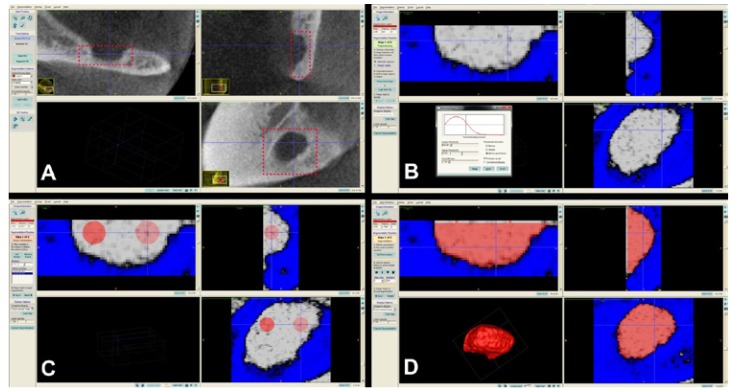
In ITK-SNAP software, region of interest was determined in a square area in all three planes of views (A). “Segment 3D” tool was used and same threshold level with the original software (Ez3D2009) was determined (B). Then the reference points were selected on the cavity for semiautomatic segmentation (C). When the “Start segmentation” tool was used, the software automatically segmented the cavity starting from the reference points using the contrast differences on the greyscale images. Thus the 3D model and the volume in mm3 were obtained with the software (D).

When measuring volumes in ITK-SNAP software, the regions of interest were determined in square areas selecting the most exterior point of the concavities in all three planar views (coronal, axial and sagittal). The “Segment 3D” tool was used and an appropriate threshold level was adjusted. Then, the reference points were selected on the cavity for semiautomatic segmentation. When the “Start segmentation” tool was used, the software automatically segmented the cavity starting from the reference points using the contrast differences on the greyscale images. Thus the 3D model and the volume in mm3 were obtained with the software (Fig. 1).

All of the measurements were made by 2 observers (experienced in image segmentation) blinded to each other over a one-month period. 2 maxillofacial radiologists separately evaluated 14 CBCT images on a computer monitor (Philips 273EQH 27-inch LED monitor with 1920 × 1080 resolution), under ambient lighting conditions. The examiners were left to choose their own threshold settings based on the best visualization of the cavities. Intra-observer reproducibility was assessed by having the observers reevaluate 5 randomly selected CBCT images after an interval of one week.

**Figure 2:**
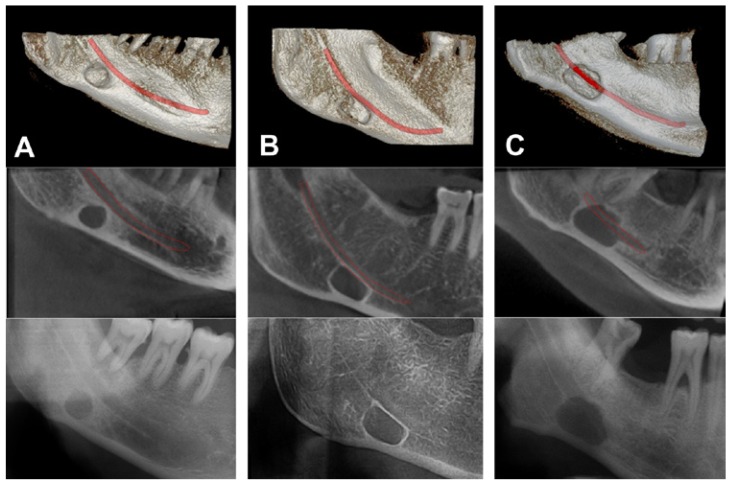
This figures show the SBCs relation to mandibular canal on CBCT images (3D and sagittal view) and panoramic radiographs. Three types of relation were observed. A) Mandibular canal is separated from the SBC. B) Mandibular canal is in contact with SBC. C) Mandibular canal goes through the SBC.

Following the volume measurements, SBCs relation to mandibular canal was also examined and three types of relation were observed (9) (Fig. 2):

Type 1: Mandibular canal is separated from the SBC.

Type 2: Mandibular canal is in contact with SBC.

Type 3: Mandibular canal goes through the SBC.

All the obtained data were entered in the Statistical Package for Social Sciences Program (SPSS 11.5). A Non-parametric Kruskal-Wallis test was used to compare inter-reliability of volumetric measurements between observers (*p*<0.05). Cohen’s Kappa and Cronbach’s Alpha tests were used to test intra-observer agreement. Also correlation between lesions volume and proximity with mandibular canal was evaluated using Pearson’s correlation coefficient.

## Results

There were 12 males and 2 females who had SBC in this study (age range: 37-73, mean age: 55.3 years).

Cohen’s Kappa and Cronbach’s alpha values showed excellent intra-observer agreement (kappa > 90 and alpha > 93) for both observers. Inter-reliability of volumetric measurements between observers was shown in  Table 1. There was no significant difference between observers for volume measurements (*p* > 0.05). The total volume of SBC ranged from 160 mm3 to 520 mm3 (mean: 361.7 mm3) ( Table 2). All the SBCs were posterior lingual variant. Bilateral presentation was not noted. Whereas 14 patients had unilateral SBC; 8 (54.1 %) were left and 6 (45.9 %) were on the right side. According to relationship of SBC with mandibular canal, most SBCs were Type 1 (64.3 %) followed by type 3 (21.4 %) and type 2 (14.3 %). Pearson correlation coefficient shows a positive correlation between lesions volumetric size and relation with mandibular canal (pearson correlation = 0.54, sig < 0.05).

**Table 1:**
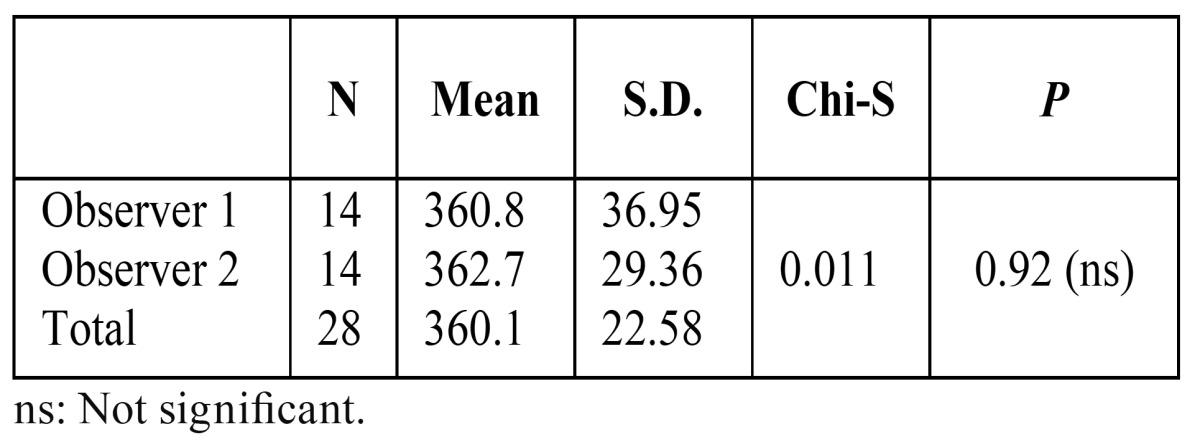
Inter-reliability of volumetric measurements between observers.

**Table 2:**
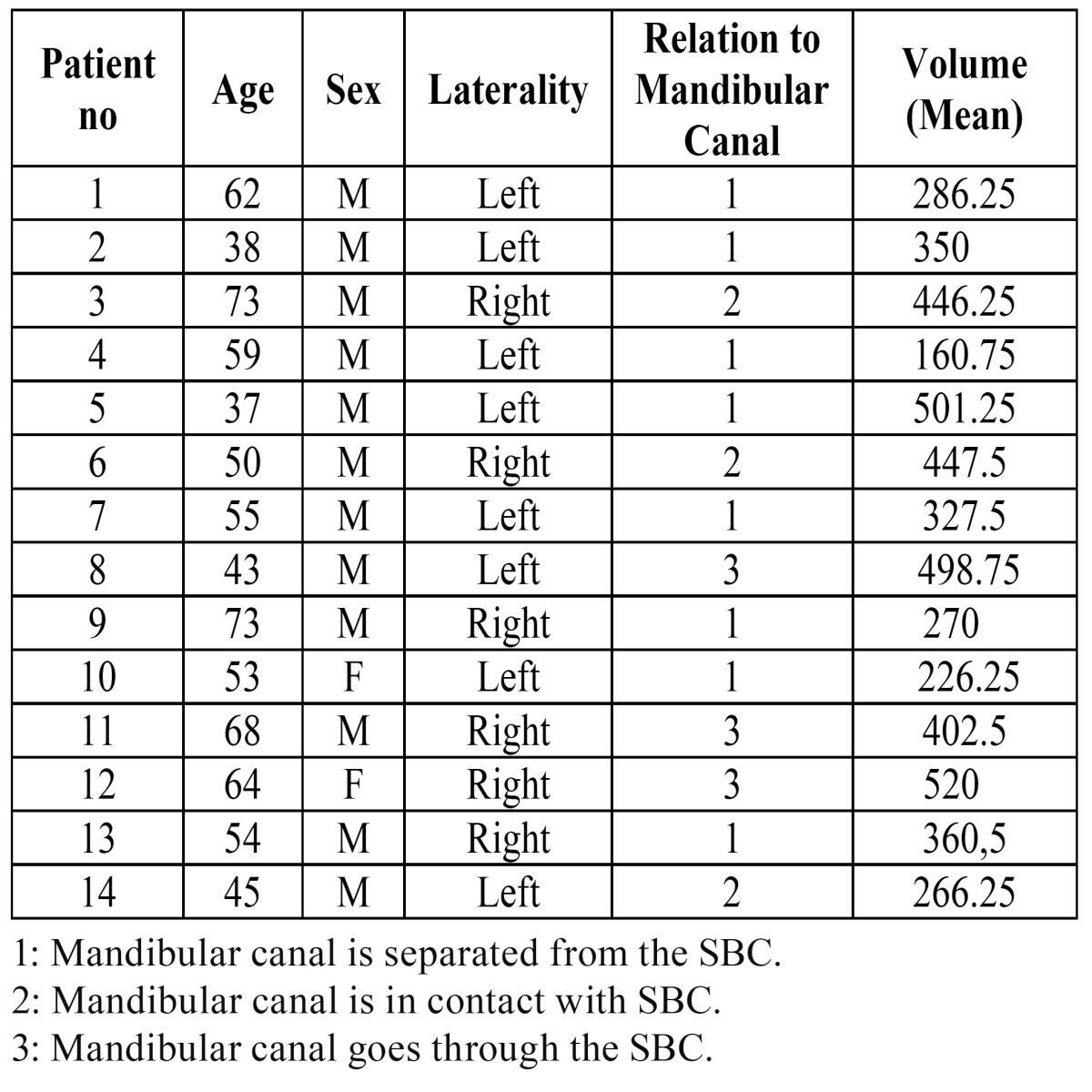
SBC characteristics of 14 cases.

## Discussion 

The aim of the present study was to evaluate the efficacy of CBCT in volume measuring using Stafne Bone Cavities’ (SBC) as an example. No significant differences were found between observers for volume measurements and the total volume of SBC found to be ranged from 160 mm3 to 520 mm3 (mean: 361.7 mm3). In the previous studies diameter and depth of the cavities were measured and it was reported that SBCs sizes varied 1 to 3 cm in diameter (10). The depth and width of the concavities averaged 7, 9 and 16, 3 mm respectively (11). To the best of our knowledge, this is the first study measuring volumetric sizes of SBCs.

Volumetric measurements can be performed using various PC programs such as Mimics, Dolphin, ITK-Snap etc. Weissheimer *et al* compared the accuracy of 6 different imaging softwares (Mimics, ITK-Snap, OsiriX, Dolphin3D, InVivo Dental, Ondemand3D) for 3-dimensional analysis of the upper airway and found that all 6 imaging software programs were reliable but had errors in the volume segmentations of the oropharynx. Mimics, Dolphin3D, ITK-Snap, and OsiriX were similar and more accurate than InVivo Dental and Ondemand3D for upper airway assessment (12). Additionally Alhowalia *et al*. tested the accuracy of CBCT for volumetric measurement of simulated periapical lesions and concluded that CBCT is an accurate means of making volumetric measurements of artificially created cavities within bone with the use of appropriate software and training (2).

Currently, there are few studies confirming the accuracy of CBCT to measure lesions in bone volumetrically (5). Previous studies have employed methods using calibration cubes, spherical phantoms of known size or calculating volume from manual linear readings (4,13). However it is uncertain whether this same method would work for lesions of irregular shape. Alhowalia *et al*. used manual tracing of simulated bone lesions on bovine bones and found that measurements were highly accurate and inter-observer variability to be relatively low. They emphasized that manual tracing is more time consuming than software guided segmenting and suggested that it would be beneficial to study further the accuracy of software-guided segmenting (2).

When using automated segmenting method it is important to select appropriate threshold level and borders of the lesion being segmenting. Otherwise the volume of the lesions may be measured incorrectly. Hence the observers should get training by a reference standard examiner (14). In the present study observers were experienced in image segmentation and got training before.

**Figure 3:**
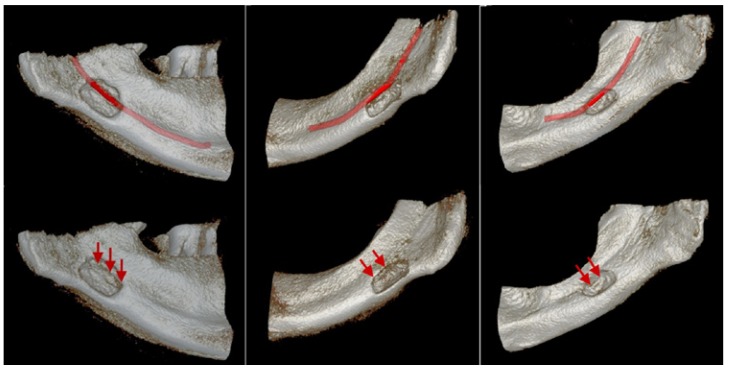
3D images of 3 cases that mandibular canal is going through the cavity.

The typical radiographic presentation of a SBC is of an elliptical, homogeneous radiolucency with a well-defined border sited below the inferior alveolar canal, often involving the lower border of the mandible. Extension of the cavity above the inferior dental nerve was rarely reported (9). Oikarinen and Julku found three cases immediately adjacent to the mandibular canal in their study of 10000 panoramic radiographs (15). In the present study we found that mandibular canal was going through the cavity in 3 cases (Fig. 3) and in contact with the cavity in 2 cases. Also larger lesions were found to be involved with mandibular canal more likely than smaller lesions. Uemura refers to a case in Japanese literature where on the radiograph, the neurovascular bundle ran centrally, but at surgery a completely closed cavity without a lingual defect was found (16). A key to anatomic feature of SBC is that the mandibular concavity always is open on its lingual face (17). Therefore this case cannot be diagnosed as a SBC due to lack of lingual defect. The advanced imaging techniques become important in such cases. The panoramic and the oblique lateral radiographs are insufficient to verify the integrity of the bone margins (9). Hence CT, MRI and sialography techniques have been used to achieve a final diagnosis of SBC (18). Sisman *et al*. reported that CT scans are usually sufficient for definitive diagnosis of SBCs (19). However Segev et al. mentioned that CT should be supported with MRI to identify the content of the cavity (20). In addition, Smith et al. reported that increased ionized radiation exposure and possible contrast allergy are disadvantages of CT and they concluded that MRI is the most useful diagnostic tool for detecting the content and extent of SBC (21). However, its high cost and relatively long imaging time limit the routine use of MRI. Sialography is also a diagnostic technique that can be used to determine whether glandular tissue exist in the cavity (22). On the other hand this procedure is invasive and uncomfortable for patients. Additionally allergy - like hypersensitivity reactions have been observed after use of X-ray contrast media. Serious events such as angioedema, subglottic oedema, bronchospasm and allergic shock are possible (23). Also sialographic evaluation of anterior variants of SBC is relatively hard to perform owing to multiple ducts in the sublingual gland (19). Recently, CBCT with high-level spatial resolution has been used for diagnostic imaging of the oral and maxillofacial regions and various anatomical structures. Owing to its lower radiation exposure, low cost and higher speed relative to conventional CT and MRI has made CBCT a valuable tool for diagnosis of various bone lesions (24). Additionally considering the ALARA (As Low As reasonably Achievable) different FOV sizes can be selected for different radiographic modalities in many CBCT devices. Matching the FOV to the area of interest can optimize the effective dose (22 cm FOV -206.2 ?Sv, 13 cm FOV- 133.9 ?Sv and 6 cm FOV- 96.2 ?Sv. 2007 International Commission on Radiological Protection tissue-weighting factors for the i-CAT Classic CBCT machine) (25). Katz *et al*. suggested that CBCT provided detailed information about the definitive diagnosis of SBC (26). Sisman *et al*. suggested that since CBCT has low radiation dose and shows fine details and superior features in distinguishing suspicious radiolucent lesions of the mandible, it might be used for diagnosis of SBC (19).

The radiographic diagnosis of SBCs has included benign salivary gland tumours, neurogenic tumours, hemangioma, myxoma, central giant cell lesion, odontogenic cyst, simple bone cyst, ameloblastoma, fibroosseous lesions, multiple myeloma, eosinophilic granuloma and metastatic disease (27). The literature featured a case of ossifying fibroma presenting as SBC (28). Some studies showed that SBCs were not developmental lesions and continued to grow gradually and become larger over a long period of observation. In addition, superimposed pathology, such as pleomorphic adenoma, can develop in the entrapped salivary gland (29). In the present study we did not aim to observe if any alterations become in SBCs over time, however with the guidance of the volumetric measurements; changes in the volumetric dimensions can be observed through further studies. Management of SBC should be conservative; a long term radiographic follow-up is required. Surgical exploration and biopsy should only be performed when the diagnosis is uncertain or an unusually severe pathology is suspected.

In conclusion, based on the results of this preliminary study, CBCT was considered to be an effective radiographic technic for measuring volumetric sizes of SBCs. However further studies with larger sample sizes are needed to prove the usefulness of CBCT in volume measurements when reconstructive surgery or follow-up study will be performed on various bone lesions.
